# Outcomes of a US-Sino family medicine leadership program

**DOI:** 10.1186/s12909-022-03856-z

**Published:** 2022-11-14

**Authors:** Jennifer Liu, Jessica Koran- Scholl, Jenenne Geske, Jeff Harrison, Mike Sitorius, Kim Jarzynka

**Affiliations:** Department of Family Medicine, University of Nebraska Medical Center, 13325 Millard Avenue, Omaha, NE 68137 USA

**Keywords:** China, Global family medicine, Faculty development, Education/curriculum development

## Abstract

**Background and objectives:**

The purpose of this study was to determine the outcomes of a two- week US-Sino Family Medicine Leadership Symposium for medical educators from China and how participants have integrated their learning into their teaching and practice of medicine.

**Methods:**

Teaching topics emphasized principles of family medicine, teaching methods, assessment, and curriculum development. Each cohort received a wide range of practical, didactic and hands-on learning experiences. Online surveys were distributed anonymously to participants from the 2013–2019 cohorts to assess learner opinion and learner behavior change as a result of the leadership symposium. Quantitative measures assessed their level of integration of the topics into teaching and clinical practice and their satisfaction in the areas of teaching and leadership. They were also asked to provide qualitative feedback regarding incorporation of the content into their work.

**Results:**

The survey response rate was 47.6% (39/82). Respondents stated that they incorporated topics such as basic interviewing skills and information on the patient-centered medical home into their teaching in China. The most applied clinical skills they were able to incorporate into their clinical environment in China included: Breaking Bad News, Simulations Sessions with practice, One-Minute Preceptor, and Interprofessional Education.

**Conclusions:**

Results indicate that participants have demonstrated behavior changes that have led to the incorporation of the content into teaching and clinical practice. We demonstrated effectiveness of the curriculum in cultivating the teaching and practice of family medicine. The program appears to be a positive experience that has led to embracement of the roles as trainer and leader. 100% of the participants who completed the survey felt that the program improved patient confidence in their ability as a family doctor. Future assessment on barriers to their progress as teachers and leaders in family medicine would be helpful to explore.

## Background

Since 2006, China has been strengthening their primary care workforce by training family medicine doctors [1–3]. That year, a nationwide family medicine training base was organized and evaluated by China’s Ministry of Health and sought to improve care at the grassroots level by growing community health centers and the practitioners to work in them [1]. Up until then, Chinese medical colleges tended to exclude clinical practice training in family and community medicine [4, 5]. With its increasing population, burgeoning economic development, and demands for better health care, China recognized the need to adapt the “new medical model of biology-psychology-society and to meet tertiary health care needs.” [4] China continues to work toward their goal of establishing a robust primary care network, but there have been challenges in recognizing the roles and importance of family doctors as well as standardizing the content and quality of training [6, 7]. Patient satisfaction and trust have been areas of concern in China and the lack of doctor-patient communication and shared decision-making contribute to this [8, 9]. There has been a need to develop more community based primary care-oriented curricula [10, 11] and increase exposure to family medicine and family medicine role models to have a positive impact on the profession of family medicine and recruitment to the specialty [10]. In order for family medicine to have a sustainable future, trainers need practical education on how to: ensure safe and effective patient care through training, teach and facilitate learning, enhance learning through assessment, and support and monitor educational progress [12].

For the past 12 years, the University of Nebraska Medical Center (UNMC) Department of Family Medicine has partnered with universities in China to develop family medicine faculty. Medical foreign exchange programs exist [2, 5] between family medicine departments in China and the US, as well as collaborations between China and other countries, however we could not find any publications that have studied outcomes of these collaborations. Our two-week US-Sino Leadership Development Symposium was created for teaching physicians from China to travel to UNMC to build educational and leadership capacity in family medicine, foster the continued development of family medicine in China, and prepare them to address the health needs of their communities. Several exchanges have been done over the years with our faculty also traveling to China on many occasions.

## Methods

### Curriculum

After direct observation, review of the family medicine curriculum in China, and surveying the needs of the Chinese faculty, we chose to address topics that would help the Chinese faculty develop curricula to nurture principles of family medicine in their learners (comprehensive, compassionate, collaborative, coordinated, patient-centered care), particularly the humanistic aspects that are characteristic of family medicine. Initially, these concepts were new and unpracticed. Authors of a Lancet article on the quality of primary care in China appear to be in agreement and recommended that “training should prepare students to work in interprofessional teams, and emphasis should be placed on the importance of doctor–patient communication, which for example includes empathy and shared decision making to build trust between patients and primary care providers [13].” We also recognized contrasts in how content was delivered in the US vs China. Chinese medical colleges used strict lectures and laboratory classes to deliver content [4], though over the years it was observed that PBL instruction was introduced and now has become more widely used in teaching.

Participants of our US-Sino Leadership Program completed educational sessions ranging from basic interviewing skills and population health, to physician leadership. Other topics introduced contemporary methods of teaching, assessment, curriculum development, and collaborative care. The format included inter-departmental lectures in small groups, interactive case presentation and discussion of patients, role playing, simulation training, and demonstrations of procedures. Additionally, there were shadowing opportunities to observe US family medicine physicians on the inpatient teaching service and ambulatory clinics using the strategies and techniques being taught, attendance of teaching day (resident teaching day), and presentation of a final project in which the participants demonstrated the knowledge and skills they attained and described how they would incorporate it into their teaching and/or clinical environment in China. Table 1 illustrates a typical two-week agenda for the leadership symposium.

**Table 1:** Typical US-Sino Family Medicine Symposium agenda

MONDAY	TUESDAY	WEDNESDAY	THURSDAY	FRIDAY
Welcome breakfast History of FM Program overview Standardization Final project overview Teaching procedures	Compliance training Q&A with faculty Small group teaching Welcome dinner	Simulation- cardiac arrest Course planning Behavior change	Community and rural teaching Basic interviewing Videotaped encounters Quality improvement Simulation- mega delivery Problem based learning Observership review	Inpatient observership Leadership Nursing home visit
Outpatient observership Primary care and mental health integration SMART tools Evaluation tools	Interprofessional Education Managing the angry patient Wellness Giving feedback Review videotaped encounters Project preparation	Attendance at resident teaching day Breaking bad news Population and community health Health disparities	Visit DHHS and State capital	Evaluations/ feedback Final project presentations Graduation ceremony

### Participants

Participants in the Symposium were health care professionals and administrators in China who had an interest in becoming future leaders in educational and care delivery models. Many were in the mid-career phase. Some originally worked in other specialties but were being retrained as family doctors due to China’s efforts in growing their primary care workforce. Most were already practicing family physicians or GP’s working in community health centers, where they reported seeing an average of 60 patients per day and spent approximately one day per week (8 hours) teaching.

### Measures

Our project assesses the outcomes of the two-week US-Sino Family Medicine Leadership Program. To date, seven cohorts (*n* = 82) participated from 2013 to 2019. The UNMC Institutional Review Board deemed this project exempt.

In September 2019, an online anonymous survey (translated to Mandarin) was administered to all past participants (*n* = 82) of the US-Sino Leadership Symposium regarding their experience. Using Kirkpatrick’s four level approach [14] as a model for evaluating our learner outcomes, we assessed 1) learner satisfaction, and 2) if and how they have applied their knowledge to their teaching and/or clinical environment. In addition, a question regarding improvement in interpersonal trust in the doctor-patient relationship was included.

## Results

Thirty-nine of 82 participants responded to the survey (47.6%; Table 2). The majority of respondents (64.1%) were female, the average age was 38.7 years (SD = 5.1) and they have been in practice for an average of 14.5 years (SD = 6.6). On average, they spend 26 hours per week (SD = 13.0) seeing patients and 7.9 hours per week teaching students and/or residents. Table 2 includes a description of our sample.

**Table 2:**
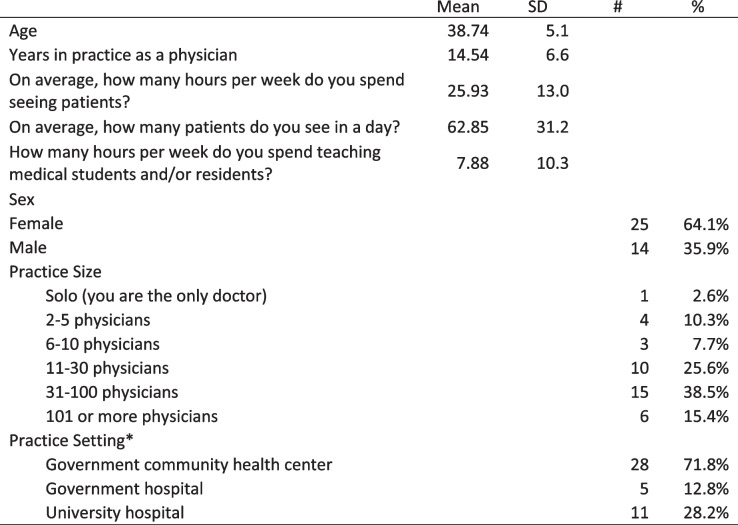
Description of sample

*Respondents could check more than one answer; sum of percents may be greater than 100%

Respondents incorporated most skills and concepts into their current teaching and/or practice. The most frequently applied teaching skills included Basic Interviewing Skills and information about Patient-Centered Medical Homes; the most applied clinical skills included Breaking Bad News, Simulations Sessions with practice, One-Minute Preceptor, and Interprofessional Education.

**Fig. 1:**
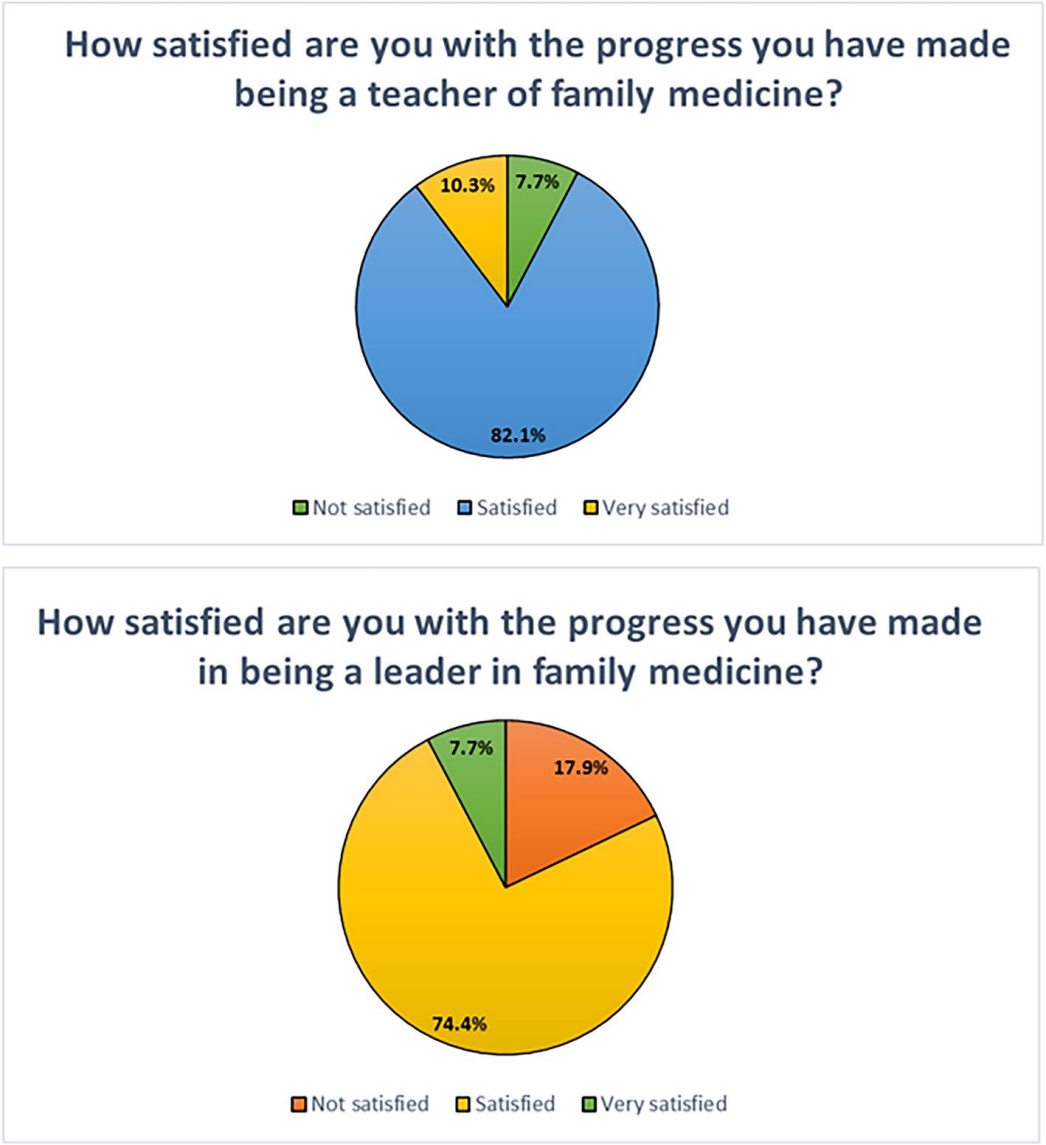
Satisfaction with progress since attending the Symposium

**Fig. 2:**
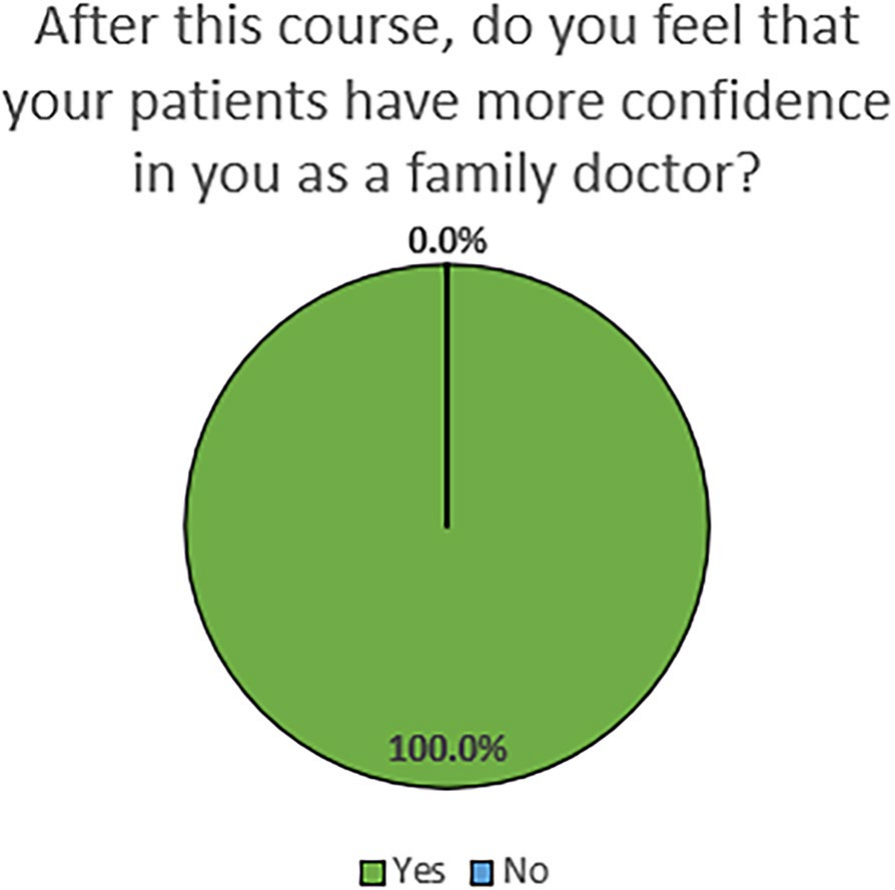
Perception of change in patient confidence since attending the Symposium

Table 3 illustrates skills that the participants were able to incorporate into teaching and clinical practice when they returned to China. Most attendees were Satisfied or Very Satisfied with their progress as a teacher and a leader of family medicine (see Fig. 1). All respondents (100.0%) felt that the program improved patient confidence in their ability as a family doctor(see Fig. 2).

**Table 3:** Skills incorporated into teaching and clinical practice

Which of the following topic areas have you incorporated into your teaching or clinical practice?	Teaching		Clinical Practice	
	#	%^a^	#	%^a^
Basic Interviewing Skills	29	74.4%	31	79.5%
Patient Centered Medical Home	29	74.4%	33	55.4%
Wellness Education	27	69.2%	31	56.9%
Giving Feedback	26	66.7%	31	79.5%
Simulation session – Procedural training with practice	26	66.7%	31	85.4%
Small Group Teaching	26	66.7%	23	49.7%
Problem Based Learning	24	61.5%	26	66.7%
Developing SMART Goals	23	59.0%	25	64.1%
Course Planning	23	59.0%	20	56.9%
Observerships – Time in clinic and inpatient setting	22	56.4%	32	82.1%
Behavior Change	22	56.4%	33	84.6%
Community and Rural Teaching	21	53.8%	22	64.1%
Nursing Home visit/Geriatrics	20	51.3%	28	71.8%
Breaking Bad News	20	51.3%	30	90.0%
Evaluation Tools	19	48.7%	25	64.1%
Quality Improvement	19	48.7%	29	74.4%
Population Health	19	48.7%	23	69.2%
Working with the Angry Patient	18	46.2%	33	84.6%
One Minute Preceptor	13	33.3%	19	85.4%
Interprofessional Education	13	33.3%	20	85.4%

^a^Respondents could check more than one answer; sum of percents may be greater than 100%

## Discussion

The US-Sino Leadership Symposium was well-received, and participants incorporated their learning into their teaching and/or clinical practice. It appears to be a positive experience that has led to participants’ embracement of their roles as trainer and leader and improved patient confidence in their ability.

### Limitations

One obvious limitation we encountered was language. The Chinese health care providers attending this Symposium had limited working proficiency of the English language. The curriculum was delivered in English with synchronous translation into Mandarin. It is unclear how much our visitors were able to comprehend, but they were able to present their final projects in English without difficulty.

Additionally, due to participant feedback, minor changes in curriculum occurred with each cohort, so there was some inconsistency from year to year. Participants may not have been able to implement some concepts in their respective educational or clinical environments.

Our response rate was low at 47.6%. This may be due, in part, to difficulty locating participants; in some cases, surveys were sent up to 6 years following their participation in the Symposium. Additionally, there are biases that are inherent in all survey research. Participants who had satisfying experiences may also have been more likely to respond to the survey. Social desirability bias may lead to self-reported outcomes that are less reliable/more positive than objective observations. Furthermore, participating physicians’ perceptions of their patients’ confidence may include participants’ own confidence in their abilities. Because of these biases, results should be interpreted carefully.

### Next steps

Continued follow-up with graduates will allow us to obtain updates on goal development and achievements including progress in community engagement, communication/interviewing skills, doctor-patient relationship, collaboration, and patient care outcomes.

## Conclusion

We anticipate that China’s health care reforms to train family physicians will take place with greater urgency due to the COVID pandemic. In October 2020, we adapted our curriculum to a webinar format which was attended by over 1500 medical professionals in China. Future innovation in digital learning may advance family medicine physician training and support and help meet the needs of growing their primary care workforce.

The goal of our Family Medicine Leadership Development Symposium was to provide scholars a knowledge base and skill set in teaching, curriculum, assessment, and leadership while highlighting the core strengths of family medicine - comprehensive, compassionate, collaborative, coordinated, patient-centered care. This will help define family medicine as an essential specialty in China and lead to improved access to care, quality of care, and reduced costs.

Feedback has been overwhelmingly positive and demonstrates how an immersive two-week experience can have positive impacts and lead to change in thinking, teaching, and patient care for Chinese family medicine physicians.

## Data Availability

The datasets used and/or analyzed during the current study are available from the corresponding author on reasonable request. The datasets generated and/or analysed during the current study are available in the Harvard Dataverse repository: Outcomes of a US-Sino Family Medicine Leadership Program - Jenenne Geske Dataverse (harvard.edu).
